# Development of risk prediction model for major amputation in patients with diabetic foot

**DOI:** 10.1186/s12902-026-02215-z

**Published:** 2026-03-05

**Authors:** Qingwei Lu, Xiaolu Wei, Wangao Zhang, Jun Wang

**Affiliations:** 1 Department of Pain Medicine, First Affiliated Hospital of Anhui University of Chinese Medicine, Hefei, Anhui 230031 China; 2 College of Chinese Medicine, Anhui University of Chinese Medicine, Hefei, Anhui 230038 China; 3 Department of Ulcers and Peripheral Vascular Surgery, First Teaching Hospital of Tianjin University of Traditional Chinese Medicine, Tianjin, 300193 China

**Keywords:** Diabetic foot, Major amputation, Prediction model, Nomogram

## Abstract

**Purpose:**

A risk prediction model was developed to predict the risk of major amputation in patients with diabetic foot ulcer (DFU) on admission, and instruct patients to prevent and control early, and guide doctors to make clinical decisions.

**Patients and methods:**

We used data from the Electronic Medical Record (EMR) database of the First Teaching Hospital of Tianjin University of Traditional Chinese Medicine from February 2014 to July 2020. DFU patients were divided into major amputation group and non-major amputation group, and nested case-control study method was used to identify case group and control group. The results of the first laboratory tests, imaging examinations, complications and other information of DFU patients at admission were collected, and initial predictive variables were selected. Logistic regression and LASSO regression in R software were used to develop a clinical prediction model for DFU patients with major amputation, which was displayed in the form of nomographs, and the model was evaluated by internal validation.

**Results:**

A total of 3654 patients were diagnosed as DFU, 695 patients were included in the study on the development of risk prediction model of DFU major amputation, 139 patients in the case group and 556 patients in the control group. 9 variables (WBC, Hb, ALB, Wagner grade, amputation history, smoking, ABI < 0.4, ulcer duration > 1 month, HbA1c) screened by logistic regression and LASSO regression were used as predictors of major amputation in DFU patients. The internal validation showed that the C index adjusted by Bootstrap method was 0.91 (95% CI, 0.894–0.943), the average absolute error of the prediction model for drawing the calibration curve was 0.01, and the brier score was 0.08.

**Conclusions:**

The clinical risk prediction model of major amputation in DFU patients developed in this study has good discrimination and calibration, can accurately predict the outcome events, can be used as an effective tool to guide doctors to make clinical decisions, and enrich and improve the content of DFU prevention and control work, but the promotion and use of the model still needs further verification of external data.

**Clinical trial number:**

Not applicable.

## Introduction

The prevalence of diabetes mellitus increased recently, which has led to an increase in related complications. Diabetic foot ulcer (DFU) is a severe chronic complication that affects diabetic patients’ feet and is a deep tissue lesion linked to peripheral vascular disease (PVD) and lower limb neuropathy. The main adverse outcome of DFU is amputation and death, and DFU is the primary reason for non-traumatic lower extremity amputation (LEA) [1]. DFU accounts for approximately 85% of the more than one million diabetic patients who undergo non-traumatic LEA per year [2]. Globally, one patient is amputated every 30 s because of DFU [3]. It has been reported that the amputation rate among DFU patients in China is 5.1% [4], while in some parts of Africa is as high as 52% [5]. Additionally, patients who have had a previous limb amputated have a 5-year mortality rate of above 50% [6, 7]. The limb functions and quality of life of patients after amputation further declined, especially for patients with major amputation, whose injury scores were significantly higher than those of patients with minor amputation, which caused huge psychological pressure and economic burden to patients [8, 9]. Therefore, the early prevention and control of DFU and the identification of risk factors for major amputation are of great significance for improving the quality of life, reducing the amputation rate and mortality, and reducing medical costs. The development of a prediction model for the occurrence of major amputation in DFU patients can fully evaluate the patients, and thus help doctors to carry out doctor-patient communication and clinical decision-making.

Limb salvage is an important topic in clinical work, and it is also the common goal of doctors and patients. However, when DFU progresses to an extremely severe stage and the patient’s general condition does not permit limb salvage, limb salvage should be abandoned to prioritize life preservation, improve the patient’s quality of life, and maximize their benefits. Generally, doctors decide whether to perform major amputation according to clinical symptoms and specific conditions of patients, and also consider the wishes of patients and their families. At present, there is still no objective and effective clinical tool to predict the risk of major amputation in DFU patients. The purpose of this study is to develop a scientific clinical risk prediction model, which can objectively and quantitatively predict the risk of major amputation in DFU patients, and provide a practical prediction tool for clinicians to guide clinical practice. The prediction model is used to fully evaluate the patients, identify the high-risk population of large amputation, and take the best intervention measures.

## Patients and methods

### Criteria for inclusion of patients

**Fig. 1:**
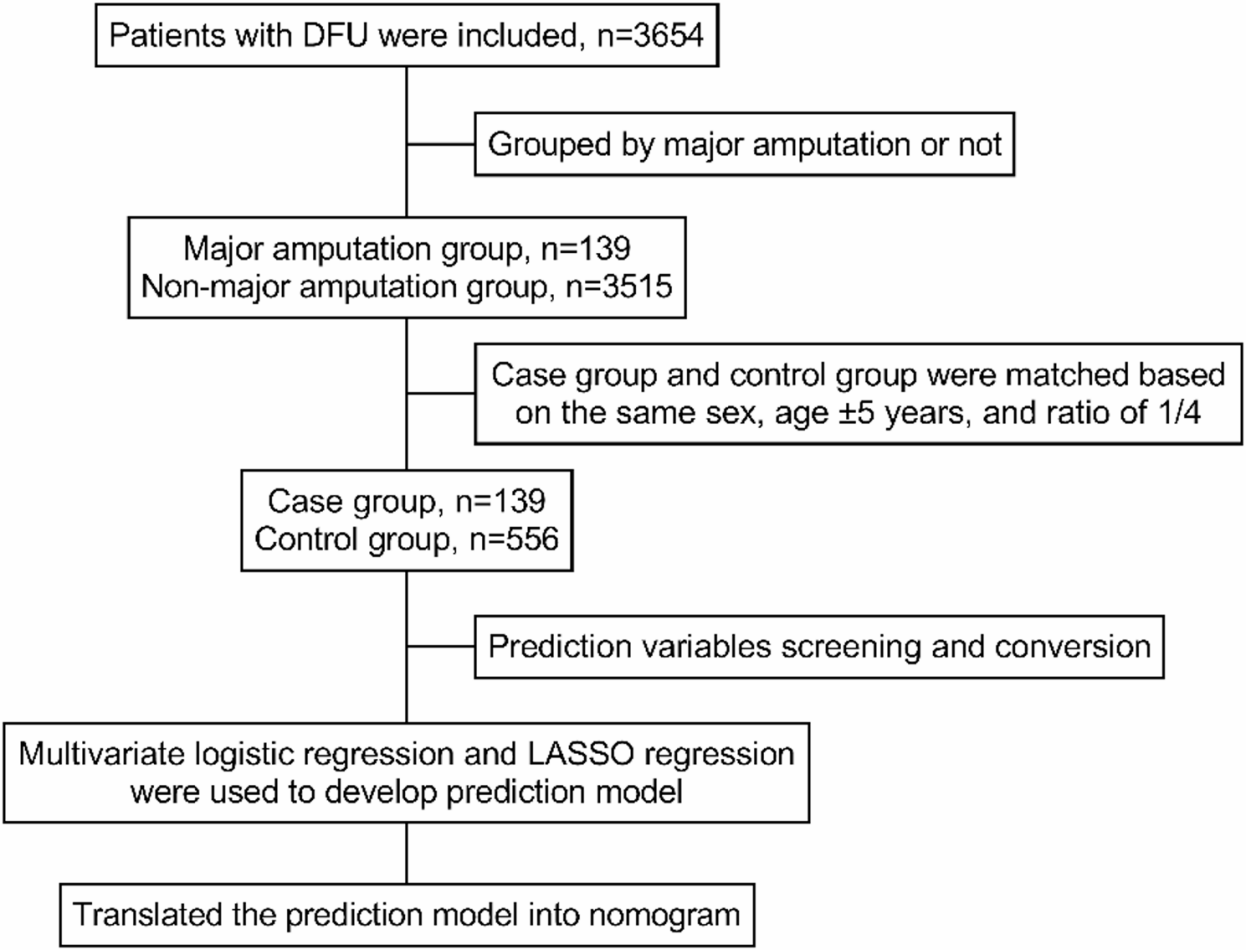
Study design summary

We extracted the inpatient data from February 2014 to July 2020 from the Electronic Medical Record (EMR) database of the First Teaching Hospital of Tianjin University of Traditional Chinese Medicine. The national clinical version 2.0 disease diagnostic code (ICD-10) was used as a query tool, in which the diagnostic code of DFU was E14.500 × 050. All data were exported, then the patients’ data were sorted, outcomes were followed up, and a retrospective cohort study was conducted. We established inclusion criteria, which including: (a) inpatients diagnosed as DFU; (b) the time of positive outcome event (major amputation) during follow-up was within 1 month after admission; (c) the first laboratory test and imaging examination were completed within 3 days of admission; (d) all clinical materials and data of the included patients were accurate and without missing. According to whether the patients undergone major amputation within 1 month after admission, all patients included in the study were divided into major amputation group and non-major amputation group, major amputation refers to amputation occurring above the ankle joint. Nested case-control study method was used to determine the case group and control group based on the matching conditions of the same sex, age ± 5 years, and the ratio of case group to control group was 1:4. Then the clinical data of the two groups were statistically analyzed (Fig. 1).

### Predictive variables and outcome events

The demographic characteristics of the patients, the results of the first laboratory test and imaging examination on admission and the combined basic diseases were sorted out. We determined the potential predictive variables of major amputation based on literature, clinical application effect and expert opinions to ensured that these predictive variables had good predictive ability in the prediction model. By the above methods, a total of 22 candidate variables were included in the study (Table 1). The DFU patients underwent major amputation surgery within 1 month after admission was defined as a positive outcome event, and major amputation was confirmed based on the patient’s hospitalization surgical records.

**Table 1:** Baseline characteristics of participants in the case group and control group

Predictor variable	Case group (*n* = 139)	Control group (*n* = 556)	*P*-value	Statistic
Age, years	65.27 ± 10.56	65.68 ± 11.00	0.687	-0.403
Sex			1.000	< 0.001
Male	105 (75.5%)	420 (75.5%)		
Female	34 (24.5%)	136 (24.5%)		
Smoking	94 (67.6%)	285 (51.3%)	0.001	12.014
Diabetes duration, years			0.001	13.301
< 10	17 (12.2%)	129 (23.2%)		
10~20	104 (74.8%)	324 (58.3%)		
> 20	18 (12.9%)	103 (18.5%)		
Ulcer duration > 1 month	90 (64.7%)	267 (48.0%)	< 0.001	12.454
MDR bacterial infection	79 (56.8%)	337 (60.6%)	0.416	0.660
Wagner grade			< 0.001	33.25
3	15 (10.8%)	149 (26.8%)		
4	64 (46.0%)	288 (51.8%)		
5	60 (43.2%)	119 (21.4%)		
ABI < 0.4	85 (61.2%)	95 (17.1%)	< 0.001	112.506
Amputation history	58 (41.7%)	119 (21.4%)	< 0.001	24.198
WBC, ×10^9^/L	12.8 (9.9, 18.9)	8.4 (6.5, 11.2)	< 0.001	-9.620
ALB, g/L	28.91 ± 4.56	33.81 ± 5.12	< 0.001	11.06
Hb, g/L	96.03 ± 19.16	113.12 ± 20.62	< 0.001	8.826
HbA1c, %	8.60 (7.45, 9.80)	7.50 (6.50, 8.80)	< 0.001	-6.063
D-dimer _ log	-0.19 ± 0.70	-3.56 ± 0.77	< 0.001	-4.714
Fib(g/L)	6.38 ± 1.79	5.36 ± 1.80	< 0.001	-5.993
APTT(s)	36.32 (29, 40)	32.31 (27, 36)	< 0.001	-5.003
Comorbidities				
Hypertension	82 (59.0%)	363 (65.3%)	0.167	1.193
Hyperlipidemia	17 (12.2%)	53 (9.5%)	0.345	0.894
CAD	95 (68.3%)	289 (52.0%)	0.001	12.048
PAD	111 (79.9%)	376 (67.6%)	0.005	7.931
Cerebral infarction	41 (29.5%)	183 (32.9%)	0.441	0.595
Renal insufficiency	20 (14.4%)	106 (19.1%)	0.220	1.638

### Diagnostic and definition criteria

Generally, amputation above the ankle is considered major amputation, whereas amputation below the ankle is considered minor amputation. Ankle brachial index (ABI) < 0.4 is defined as severe ischemia of lower limbs. History of amputation is defined as major or minor amputation prior to hospitalization. Multi-drug resistant (MDR) bacterial infection is defined as resistance to three or more antibiotics simultaneously. These drug-resistant bacteria mainly include methicillin-resistant Staphylococcus aureus, pan resistant Pseudomonas aeruginosa, ESBL producing Escherichia coli and Klebsiella pneumoniae, pan resistant Acinetobacter baumannii and Enterococcus faecalis, etc. Coronary artery disease (CAD) and peripheral arterial disease (PAD) are diagnosed by Doppler ultrasonography and arteriography, the diagnostic criterion is that the degree of stenosis in the vascular lumen of the lesion site exceeds 50%. Patients with baseline creatinine > 1.5 mg/dl or glomerular filtration rate < 60 ml/min were defined as having chronic renal insufficiency (CRI).

### Sample size calculation

Referring to the rough estimation method of sample size of multivariate logistic regression, the principle of EPV (events per variable) requires that the EPV should be more than 10, that is, the number of positive events in the results should be 10 times more than the predictive variables in the model. We initially set that there were 10 predictive variables in the model, and the number of major amputations for positive outcome events should be more than 100 cases.

### Statistical analysis

The data were entered into Microsoft Excel software and imported into R software for analysis. The mean, standard deviation (SD), percentage and rates of the data were analyzed using descriptive statistics. Nonlinear continuous variables were converted into linear variables or categorical variables. The difference between two groups for each variable was analyzed by univariate logistic analysis. Multicollinearity and interaction were checked to confirm each variable was independent. Multivariate logistic regression analysis was used to estimate the odds ratios (ORs) and corresponding 95% confidence intervals (95% CI) and to preliminarily determine the full model. LASSO regression was used to screen the prediction factors finally included in the model, and the final prediction model was developed, which was displayed in nomographs. Bootstrap resampling method was used for internal verification of prediction model, receiver operating characteristic (ROC) curve and calibration plot were drawn to assess discrimination of the risk prediction model. The final model discrimination is determined by using the concordance statistic (C-statistic). The C-statistic value of 0.7 is considered clinically useful, and > 0.8 is considered excellent [10]. The R language software packages used mainly include “tidyverse, haven, car, gtsummary, ggplot2, rms, glmnet, MASS, pROC, plotROC, etc”. P-value < 0.05 was considered statistically significant.

## Results

### Clinical characteristics

A total of 3654 patients in the entire cohort were included in the final study. Among them, 139 patients (3.8%) underwent major amputation, including 105 males and 34 females, who were designated as the case group. A total of 3515 patients (96.2%) had no major amputation event (control group). With the same sex, age ± 5 years as the matching condition, the case group and the control group were matched according to 1:4, and finally 556 cases were included as the control group. There were significant differences in 16 variables (sex, smoking, diabetes duration, ulcer duration > 1 month, Wagner grade, ABI < 0.4, previous amputation history, WBC, ALB, Hb, HbA1c, D-dimer _ log, Fib, APTT, CAD, PAD) between the two groups (Table 1).

### Full model and variable screening

All variables were included to preliminarily determine the full model. The “glm” function was used to draw the parameter table of the full model, there were 22 variables in the full model (Table 2). We identified potential risk factors for major amputation using LASSO regression to complete variables selection. When lambda value was 0.05277, there were 9 variables in the model, and each variable included at this time was an independent risk factor for major amputation in DFU patients. At the same time, clinical medical experts of this specialty were invited to evaluate the model, and believed that the selection of variables were reasonable and the number of variables was appropriate, which the model could be used as a clinical prediction tool. Finally, the results showed that WBC, Hb, ALB, Wagner grade, amputation history, smoking, ABI < 0.4, ulcer duration > 1 month, HbA1c were included in the final risk prediction model, and each of the predictor variable was independent, mutually blind and unaffected.

**Table 2:** Parameters of each predictor variable in the full model

Predictor variable	OR	95% CI	*P*-value
Age, years	1.01	0.98, 1.04	0.6
Sex			
Male	-	-	
Female	0.59	0.29, 1.16	0.14
Smoking	2.99	1.61, 5.72	< 0.001
Diabetes duration, years			
< 10	-	-	
10~20	1.03	0.53, 1.98	> 0.9
> 20	0.44	0.18, 1.07	0.078
Ulcer duration > 1 month	2.75	1.48, 5.29	0.002
MDR bacterial infection	0.45	0.24, 0.82	0.010
Wagner grade			< 0.001
3	-	-	
4	1.72	0.73, 4.34	0.2
5	3.02	1.16, 8.33	0.027
ABI < 0.4	16.5	8.70, 33.1	< 0.001
Amputation history	2.43	1.31, 4.57	0.005
WBC, ×10^9^/L	1.13	1.08, 1.20	< 0.001
ALB, g/L	0.88	0.81, 0.95	< 0.001
Hb, g/L	0.96	0.94, 0.98	< 0.001
HbA1c, %	1.47	1.24, 1.75	< 0.001
D-dimer _ log	0.77	0.50, 1.18	0.2
Fib(g/L)	0.93	0.76, 1.13	0.5
APTT(s)	1.03	1.00, 1.07	0.074
Comorbidities			
Hypertension	0.96	0.51, 1.83	0.9
Hyperlipidemia	1.90	0.69, 5.14	0.2
CAD	3.72	1.94, 7.37	< 0.001
PAD	1.02	0.50, 2.08	> 0.9
Cerebral infarction	0.83	0.44, 1.55	0.6
Renal insufficiency	0.53	0.21, 1.29	0.2

### The final risk prediction model

**Fig. 2:**
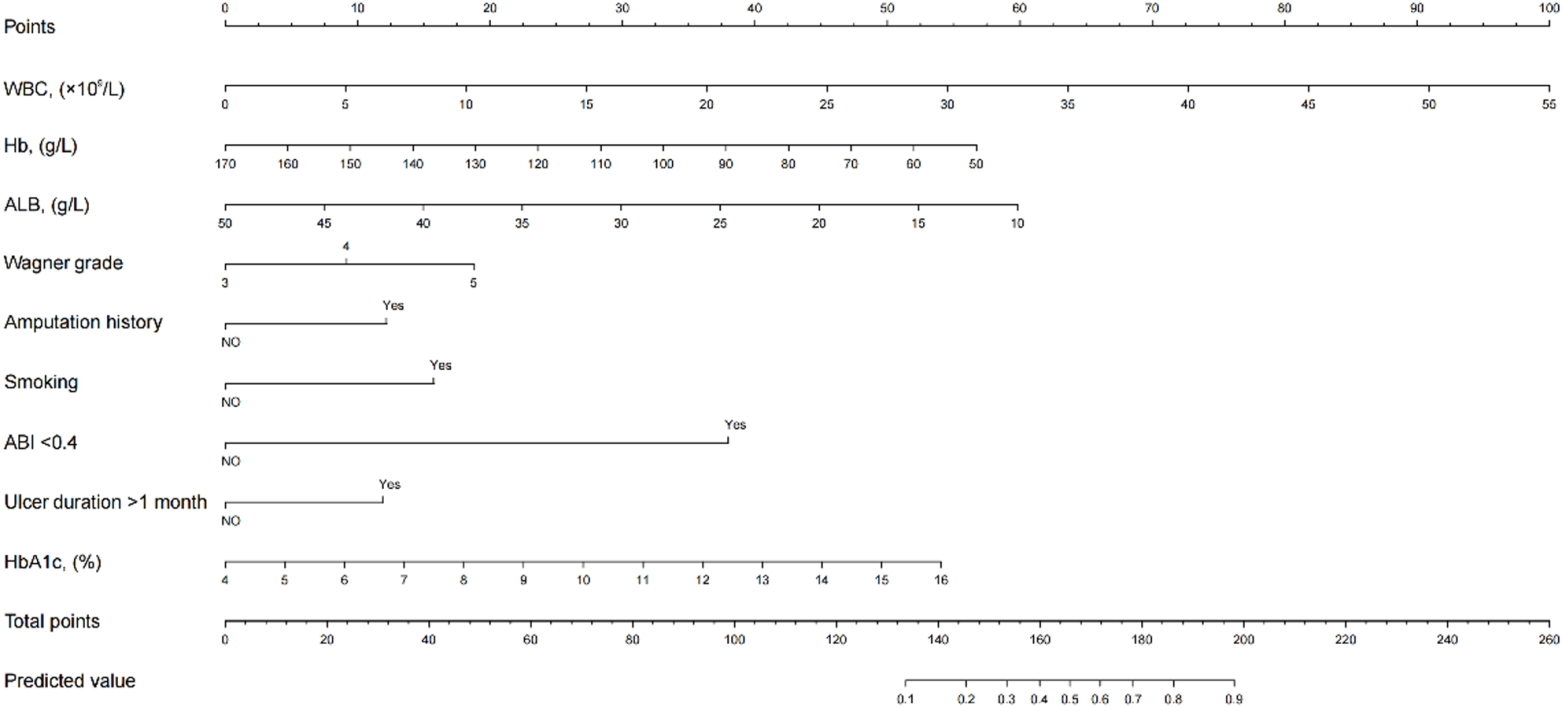
The nomogram for the prediction of the probability of major amputation in DFU patients

These predictor variables by LASSO regression and the occurrence of major amputation were taken as independent variables and dependent variables respectively, and the final model was refitted by logistic regression (Table 3). The model for major amputation was translated into a nomogram by using the “lrm” function in the R software for the ease of clinical application (Fig. 2). The instruction of nomogram: make a vertical line on the horizontal axis of each predictor variable of the patients, corresponding to the specific points on the “points” horizontal axis, add all the points of the predictor variables to get the total points, and make a vertical line downward. The value on the horizontal axis of the corresponding “prediction value” is the risk prediction value of major amputation of the DFU patients. For example, if a DFU patient’s WBC is 22.15 × 10^9^/L, Hb is 120 g/L, ALB is 35 g/L, Wagner grade 5, no amputation history, but smoking history, ABI < 0.4 and ulcer duration > 1 month, HbA1c is 7.6%, then the total points of the patient is 40 + 23+22 + 19+0 + 16 + 38 + 12+16 = 186, and the predicted value of the nomogram is about 78%.

**Table 3:** Parameters of predictor variable in the final risk prediction model

Predictor variable	OR	95% CI	*P*-value	Predictor variable
WBC, ×10^9^/L	1.13	1.08, 1.19	< 0.001	0.1240
Hb	0.97	0.95, 0.98	< 0.001	-0.0322
ALB	0.90	0.85, 0.96	0.002	-0.1020
Wagner grade				
3	-	-		
4	1.87	0.86, 4.30	0.130	0.6242
5	3.59	1.54, 8.85	0.004	1.2795
Amputation history				
No	-	-		
Yes	2.29	1.30, 4.06	0.004	0.8286
Smoking				
No	-	-		
Yes	2.92	1.69, 5.19	< 0.001	1.0731
ABI < 0.4				
No	-	-		
Yes	13.3	7.58, 24.4	< 0.001	2.5899
Ulcer duration > 1 month				
No	-	-		
Yes	2.25	1.28, 4.03	0.006	0.8100
HbA1c	1.36	1.17, 1.58	< 0.001	0.3072

### Internal validation and evaluation of risk prediction model

**Fig. 3:**
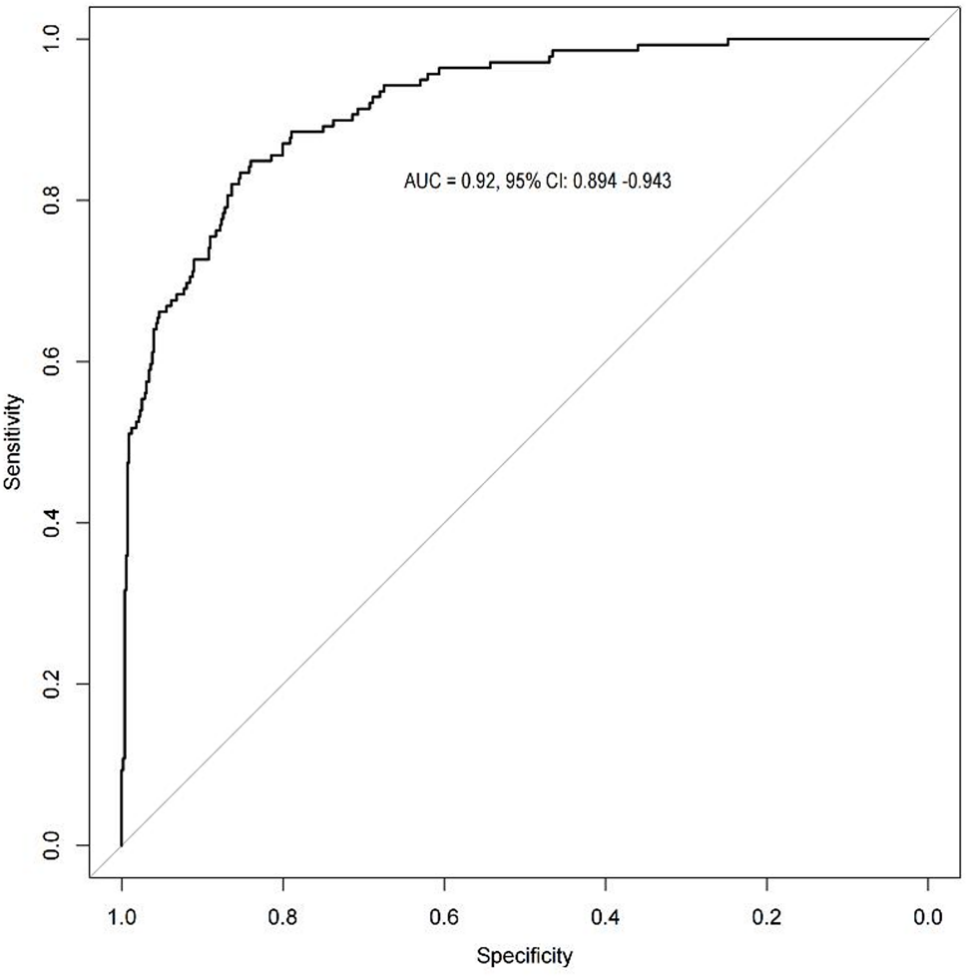
ROC curve of risk prediction model

**Fig. 4:**
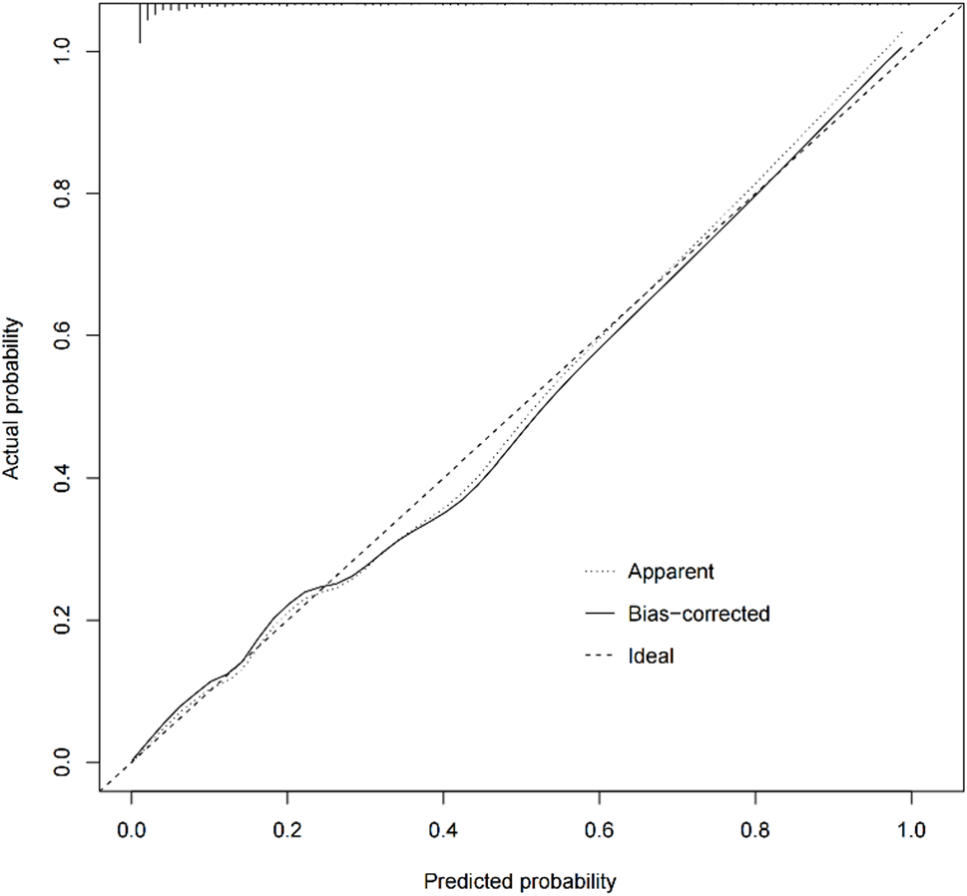
Calibration curve of risk prediction model. Notes: … Apparent was the performance of the model in the original data, — Bias corrected was the performance of the model after resampling and internal verification, --- Ideal was the most ideal model performance

The C-statistic calculated by R software was 0.92, that was, the area under the curve was 0.92 (95% CI: 0.894–0.943) (Fig. 3). In order to verify the repeatability of the model development process, we conducted internal validation of the model. The “rms” package was used to conduct the enhanced bootstrap method for repeated sampling for 1000 times, the adjusted C-statistic was 0.91, indicating that the predicted results were highly consistent with the actual observation results, and the model had good discrimination. Drawn the calibration curve of the prediction model, and calculated the mean absolute error (MAE) between the model’s prediction of major amputation risk and the actual risk was 0.01, brier’s score was 0.08, indicating that the prediction model had good calibration and could accurately predict the outcome event (Fig. 4).

## Discussion

In the past literature, the risk factors of DFU major amputation have been widely studied. Clinicians can implement early interventions targeting these risk factors to reduce the incidence of major amputation. When DFU patients were hospitalized, patients and their families often asked such a question: “Doctor, can I save my leg?”. Although clinicians can answer this question based on experience, such judgments are subjective and limited by the clinician’s expertise. We used the Youden index cut-off point for this study, which had both maximum sensitivity and specificity. Moreover, the model had good calibration and discrimination, indicating that one applicative prediction tool to assess the occurrence risk of the DFU patients for major amputation. We found that WBC, Hb, ALB, Wagner grade, amputation history, smoking, ABI < 0.4, ulcer duration > 1 month, HbA1c were independently predicted an increased risk of major amputation in DFU patients. Reasonably, a good risk prediction model should be simple and applicable, contain important clinical variables that can maximize the impact on the predicted outcome and easy to be obtained by doctors in time, which is convenient for clinical workers to use. Based on this 9-item assessment, doctors are able to easily and conveniently identify high-risk DFU patients for major amputation and directly translate professional recommendation into actions, for instance, immediate major amputation, limb salvage treatment, or early intervention against risk factors.

Our study suggested that the DFU patients who had suffered major amputation had elevated inflammatory marker (WBC), more severe foot problems (Wagner grade, ulcer duration > 1 month, amputation history), reduced nutritional level (Hb, ALB), poor blood glucose control (HbA1c), severe ischemia (ABI < 0.4) and smoking in daily life. The prediction model consisted of 9 variables presented major amputation risk. The amount of WBC, an inflammatory sign for DFU, can indicate the wounds’ severity of infection, higher WBC levels were associated with a higher likelihood of major amputation, according to our study. Jiang et al. study’s findings also demonstrated a link between rising WBC levels and a higher risk of major amputation in DFU patients in China ([OR] 1.10) [11]. There is debate regarding the use of other inflammatory markers such procalcitonin, ESR, and CRP as amputation predictors [12]. However, regardless of whether they would face major amputation, DFU patients who have recently been admitted to the hospital will benefit from receiving early, sensitive anti-infective medication.

The ALB and Hb levels were discovered by studies of Aziz and Namgoong et al. to be important prognostic indicators for major amputation [13, 14]. Our study demonstrated a negative correlation between serum ALB and Hb levels and the risk of major amputation in DFU patients [15]. More oxygen molecules are delivered to target tissue as serum Hb levels rise. Additionally, serum ALB and Hb can be utilized as markers to assess the body’s nutritional condition. Low serum ALB and Hb levels impair DFU wound healing because the wound consumes substantial energy during the healing process. The significant risk of major amputation in people with DFU is unquestionably related to this disorder.

According to other investigations, the Wagner grade-based severity rating was a significant risk factor for LEA [16–18]. A prior study supported the finding that Wagner 5 enhanced the chance of major amputation by more than five times [15]. This finding was not unexpected given that the Wagner grade rose with the seriousness of the ulcer, involving wound depth, osteomyelitis, gangrene.

The amputation history covered both major and small amputations, which showed how patients with DFU developed over time [19]. A research showed that there were 24.4% of DFU patients received ipsilateral re-action within 12 months after the first amputation [20]. According to Miller’s study, individuals who undergo minor amputation were more likely to need amputation below the knee [21]. Besides, A meta-analysis that included 1,873 amputation patients came up with the finding that patients with amputation history had a 1.47-fold higher risk of re-amputation than without such a history [18]. LEA alters the biomechanics of the amputated limb and may lead to increased pressure zones and abnormalities. Patients who have had a major amputation in the past rely solely on the opposite leg for mobility, rendering that limb more vulnerable to injury, repeated ulceration, and LEA. Consequently, nursing care should be enhanced to lower the likelihood of another amputation in patients with DFU who have had an amputated limb (major or minor), prevention and control actions should be taken in beforehand, loose and comfortable shoes should be chosen to minimize the burden influence on the injured area and the physical or chemical damage to the lesion site, and so on.

Smoking was identified as a risk factor, smoking cessation was proven to be protective against DFU amputation, and smoking was linked to DFU amputation [22]. Additionally, smoking might increase the 30-day readmission rates in patients with DFU who had undergone previous LEA ([OR] 3.22 [95% CI] 1.40–7.36) [23]. Cigarette smoke contains a lot of harmful chemicals. Among them, harmful substances such as carbon monoxide, nicotine, acrolein, and oxidant compounds can aggravate vascular calcification, lead to dysfunction of vascular endothelial cells, produce oxidative stress reaction and excessive release of inflammatory factors [24]. Therefore, strict smoking cessation should be recommended for DFU patients in clinical practice.

It was well recognized that patients with diabetic foot lesions have limb ischemia as a separate risk factor for amputation. Surprisingly, Calle-Pascual’s study revealed that peripheral vascular disease affected every patient who had undergone a major amputation [25]. ABI may more accurately represent the severity of lower limb ischemia. Our earlier research revealed that patients with DFU had a 15.77-fold increased risk of major amputation when their ABI was below the threshold for mild-to-moderate ischemia, which is defined as ABI < 0.4 [15]. In addition, Faglia also discovered that if diabetes patients had severe limb ischemia, and they did not receive revascularization, then the incidence of major amputation increased significantly 30 days and 5 years later [26]. These illustrated the significance and requirement of evaluating the condition of lower limbs arteries in DFU patients. To sum up, a major amputation could be postponed or avoided with early referral to a vascular surgeon.

In our study, 1 month was taken as the cutoff point to convert the ulcer duration into a binary variable, because ulcers with a duration more than 1 month are often referred to as chronic refractory wounds, which is more clinically significant. Ugwu’s article reported that the risk of amputation increased 10 times when the ulcer duration lasted more than 1 month [27]. Similarly, The study of Uysal et al. showed that when the ulcer duration is more than 60 days, the risk of major amputation in DFU patients increases by 2.47 times [28]. This may be because the prolongation of ulcer duration will increase the risk of wound infection, leading to tissue necrosis, aggravating the disease and increasing the risk of major amputation. Therefore, we should cover the wound as early as possible to restore the integrity of soft tissue and limb function.

Previous studies found contradictory results on the impact of HbA1c levels. Yesil et al. discovered that diabetes duration and HbA1c were not risk factors for predicting overall amputations [29]. Nevertheless, our investigation found that HbA1c was a risk factor for major amputation, and Moon et al. supported this finding [30]. In diabetic individuals, HbA1c shows the average blood glucose level over the last three months. Poor blood glucose management was also shown in other research to be a potential risk for limb amputation in diabetes patients [31]. Furthermore, maintaining adequate glycemic control is crucial for DFU patients to halt the disease’s development and lower their likelihood of needing an amputation. At present, the recommended HbA1c control level should be lower than 7%.

However, the study had all the drawbacks typical of retrospective studies. Selection bias was present, and the sampled population came from a single center. The prediction model was developed with internal validation, but clinical data and external validation cohorts were still lacking.

In conclusion, we have created and validated a practical forecasting tool for predicting major amputation in DFU patients. The risk prediction model of DFU patients with major amputation obtained in this study has good discrimination and calibration, and can accurately predict the occurrence of outcome events. This model can be used as an effective tool to guide doctors to make clinical decisions that choose an individualized treatment goal and the best intervention plan for DFU patients. For example, if the prediction outcome of DFU patients is a high risk of major amputation, it may be recommended that patient immediately may undergo major amputation without hesitation to prevent disease progression and life-threatening situations, if the prediction outcome shows a low risk of major amputation, it is necessary to persist in active treatment to achieve limb salvage goals and improve quality of life. The establishment of a prediction model for major amputations in DFU patients has important clinical significance for predicting disease prognosis, promoting communication between doctors and patients, and improving work efficiency. It also enriches and improves the overall content of diabetic foot prevention and control. Nevertheless, additional research is required to increase the predictive validity and application of the risk prediction model by external validation (geographical validation).

## Data Availability

Considering the privacy of patients, if readers have similar research and want to obtain data related to the article, they can contact the corresponding author, the corresponding research data can be obtained with permission.
